# 胸腔镜肺叶切除术中转开胸83例原因分析：单手术组连续1, 350例手术总结

**DOI:** 10.3779/j.issn.1009-3419.2021.101.21

**Published:** 2021-07-20

**Authors:** 鹏 苏, 士旺 温, 明博 王, 延昭 徐, 会来 吕, 振华 李, 子强 田

**Affiliations:** 050011 石家庄，河北医科大学第四医院胸外科 The Fifth Department of Thoracic Surgery, the Fourth Hospital of Hebei Medical University, Shijiazhuang 050011, China

**Keywords:** 中转开胸, 电视胸腔镜手术, 肺叶切除术, 出血, Conversion to thoracotomy, Video-assisted thoracic surgery, Lobectomy, Hemorrhage

## Abstract

**背景与目的:**

电视胸腔镜手术（video-assisted thoracic surgery, VATS）为肺癌治疗的主流术式，本研究旨在分析单手术组连续VATS肺叶切除术1, 350例中转开胸83例的中转原因，以期对常态下胸腔镜肺叶切除术中转开胸的规律有更深层次的认识，并更好地把握中转开胸的手术时机。

**方法:**

回顾性分析2009年9月21日-2020年6月1日河北医科大学第四医院胸外科单手术组连续行胸腔镜肺叶切除术的1, 350例患者资料。其中男性773例，女性577例，年龄8岁-87岁，中位年龄61.3岁。全组良性疾病83例，肺转移瘤38例，原发性肺癌1, 229例，其中Ⅰ期: 676例，Ⅱ期: 323例，Ⅲa期: 230例。全组行左肺上叶切除术301例（22.30%），左肺下叶切除术231例（17.11%），右肺上叶切除术378例（28.00%），右肺中叶切除术119例（8.81%），右肺下叶切除术262例（19.41%），右肺中上叶切除术16例（1.19%），右肺中下叶切除术43例（3.19%）。

**结果:**

单手术组连续1, 350例胸腔镜肺叶切除术患者中有83例（6.15%）因不同原因中转开胸。良性病变的中转开胸率高于恶性肿瘤（*P* < 0.05）；病理分期为Ⅲa期的中转开胸率明显高于Ⅰ期、Ⅱ期患者（*P* < 0.05）。联合肺叶切除术的中转开胸率明显高于单肺叶切除术（*P*=0.001）；左肺上叶切除术的中转开胸率显著高于其他单肺叶切除术（*P* < 0.001）；右肺中叶切除术的中转开胸率低于其他单肺叶切除术（*P*=0.049）。中转开胸主要原因为血管损伤（38.55%）、淋巴结干扰（26.51%）、胸腔致密粘连（16.87%）；中转开胸组总体手术时间为（236.99±66.50）min，总体失血量（395.85±306.38）mL。其中淋巴结干扰组患者手术时间（322.50±22.68）min，长于其他原因中转开胸组（*P* < 0.05）；血管损伤组术中出血量（560.94±361.84）mL，多于其他原因中转开胸组（*P* < 0.05）；随着时间的推移以及经验的积累，在手术前、中、后期血管损伤例数逐步下降（*P* < 0.05）。

**结论:**

在胸腔镜手术中，肺部良性病变和较晚期恶性肿瘤有较高的手术难度和中转率。不同肺叶切除术中转开胸率不同，左肺上叶切除术中转率较高，而右肺中叶切除术中转率较低。血管损伤、淋巴结干扰、胸腔致密粘连仍是常态下胸腔镜肺叶切除术中转开胸的主要原因。中转开胸会导致手术时间延长和手术出血量增加。随着手术例数的增加，胸腔镜肺叶切除术中转开胸率有持续下降趋势，其主要原因是肺血管的处理更加成熟。

## Abstract (en)

**Backgroud and objective:**

Video assisted thoracic surgery (VATS) is the main surgical method for lung cancer. The aim of this study was to analyze the reasons for conversion to thoracotomy in 83 cases among 1, 350 consecutive cases who underwent video-assisted thoracic surgery (VATS) lobectomy by a single surgical team, in order to achieve a deeper understanding of the rules and the opportunity for conversion to thoracotomy in VATS lobectomy under normal conditions.

**Methods:**

The clinical data of 1, 350 patients who underwent VATS lobectomy between September 21, 2009 and June 1, 2020, by a single surgical team in the Fifth Department of Thoracic Surgery of the Fourth Hospital of Hebei Medical University were retrospectively analyzed. There were 773 males and 577 females, aged 8-87 years, with a median age of 61.3 years, including 83 cases of benign diseases, 38 cases of lung metastases, and 1, 229 cases of primary lung cancer. The cases with stage Ⅰ, Ⅱ and Ⅲa were 676, 323 and 230, respectively. The cases of left upper, left lower, right upper, right middle, right lower, right middle and upper and right middle and lower lobectomy were 301 (22.30%), 231 (17.11%), 378 (28.00%), 119 (8.81%), 262 (19.41%), 16 (1.19%) and 43 (3.19%), respectively.

**Results:**

In the cohort of 1, 350 consecutive patients with VATS lobectomy, 83 patients (6.15%) were converted to thoracotomy for different reasons. The conversion rate of benign lesions was significantly higher than that of malignant tumors (*P* < 0.05). The conversion rate in stage Ⅲa was significantly higher than that in stage Ⅰ and Ⅱ (*P* < 0.05). The conversion rate of combined lobectomy was significantly higher than that of single lobectomy (*P*=0.001). The conversion rate of left upper lobectomy was significantly higher than that of other single lobectomy (*P* < 0.001). The conversion rate of right middle lobectomy was significantly lower than that of other single lobectomy (*P*=0.049). The main reasons for conversion were vascular injury (38.55%), lymph node interference (26.51%) and dense adhesion in thoracic cavity (16.87%). In the conversion group, the total operation time was (236.99±66.50) min and the total blood loss was (395.85±306.38) mL. The operation time in patients converted to thoracotomy due to lymph node interference was (322.50±22.68) min, which was significantly longer than that in the other groups (*P* < 0.05). The intraoperative blood loss in patients converted to thoracotomy due to vascular injury was (560.94±361.84) mL, which was significantly higher than that in the other groups (*P* < 0.05). With the increase in surgical experience, the number of vascular injuries gradually decreased at the early stage, mid-stage and late stage (*P*=0.045).

**Conclusion:**

In VATS lobectomy, benign lung lesions and more advanced malignant tumors led to more surgical difficulties and higher conversion rate. The conversion rate was different in different lobectomy sites, with the highest in left upper lobectomy, and the lowest in right middle lobectomy. Vascular injury, lymph node interference and dense adhesion were the main reasons for conversion to thoracotomy, which led to prolonged operation time and increased blood loss. With the increasing number of surgical cases, the rate of conversion to thoracotomy in VATS lobectomy continues to decline, which may be mainly due to the more advanced treatment of pulmonary vessels.

肺癌是目前世界上最常见的恶性肿瘤之一，具有较高的发病率及病死率^[1]^，在过去的30年间，电视胸腔镜手术（video-assisted thoracic surgery, VATS）相关技术飞速发展，使其逐步取代了传统的开胸手术，广泛地应用于肺癌的外科治疗，成为了肺癌治疗的主流术式^[1, 2]^，VATS肺叶切除术的可行性、安全性及肿瘤学根治性已得到广泛认可^[3]^。然而，在进行胸腔镜手术的过程中，因为各种原因中转开胸始终是客观存在的。关于胸腔镜肺叶切除术的学习曲线^[4]^和中转开胸^[5]^等问题，多年来已经有很多报告，但这些报告仍局限于多个手术组、小样本量的研究，多数仍处于学习曲线中，缺乏单个手术组大样本研究，故不能提供常态下胸腔镜肺叶切除术中转开胸原因的理性分析。本研究回顾性分析了我院单手术治疗组连续1, 350例胸腔镜肺叶切除术中转开胸83例患者的临床资料，以期对常态下胸腔镜肺叶切除术中转开胸的规律有更深层次的认识，现总结分析如下。

## 1 材料和方法

### 1.1 一般资料

本研究为回顾性队列研究，纳入我院单手术组自2009年9月21日-2020年6月1日连续1, 350例VATS肺叶切除术患者的临床资料。其中男性773例，女性577例，年龄8岁-87岁，中位年龄61.3岁。全组恶性肿瘤1, 267例，良性疾病83例。肺部恶性肿瘤包括原发性肺癌1, 229例及肺转移瘤38例，原发性肺癌采用国际肺癌研究协会（International Association for the Study of Lung Cancer, IASLC）构建的非小细胞肺癌（non-small cell lung cancer, NSCLC）肿瘤原发灶-淋巴结-远处转移（tumor-lymph node-metastasis, TNM）分期系统进行分期，其中Ⅰ期676例，Ⅱ期323例，Ⅲa期230例。良性疾病包括：肺囊肿22例、支气管扩张15例、炎性假瘤13例、结核球9例、错构瘤8例、肺隔离症7例、中叶综合征4例、硬化性血管瘤2例、肺气肿1例、真菌球1例、畸胎瘤1例。全组行左肺上叶切除术301例（22.30%），左肺下叶切除术231例（17.11%），右肺上叶切除术378例（28.00%），右肺中叶切除术119例（8.81%），右肺下叶切除术262例（19.41%），右肺中上叶切除术16例（1.19%），右肺中下叶切除术43例（3.19%）。

### 1.2 手术方法

手术均行静脉复合麻醉，双腔插管，术中单肺通气。全组手术主要以单操作孔完成，手术切口取腋中线第7-第8肋间做一长约1.5 cm切口作为进镜口，腋前线第4肋间做一长约3.0 cm切口作为主操作口。个别手术采用纯单孔或三孔法完成，其中纯单孔切口取腋前线与腋中线第4肋间做一长约3.0 cm切口作为操作口，三孔法主要用于手术困难的病例，三孔法切口是在单操作孔切口的基础上，再取腋后线或肩胛下角线第7-第8肋间做一长约1.0 cm切口作为副操作口。主操作口由切口保护器撑开，手术操作均在电视胸腔镜下进行。镜下操作顺序与传统开胸肺叶切除基本相同，原发性肺恶性肿瘤的手术方式为肺叶切除+系统淋巴结清扫。如镜下操作遇到淋巴结粘连镜下分离困难、出血等特殊情况，将主操作口自肋间沿肩胛下角方向延长至10 cm-15 cm并应用撑开器撑开肋骨即为中转开胸。

### 1.3 统计学分析

所有数据均用SPSS 26.0软件进行统计学分析，计量资料采用均数±标准差（Mean±SD）表示，组间比较采用独立样本*t*检验，计数资料采用χ^2^检验进行比较。*P* < 0.05为差异有统计学意义。

## 2 结果

### 2.1 一般临床特征及手术病理资料比较

共纳入1, 350例VATS肺叶切除术患者，其中83例（6.15%）术中因各种原因进行了紧急中转开胸术。中转开胸组患者的性别、年龄、吸烟史、肺部合并症类型、肿瘤大小与胸腔镜手术组比较，无统计学差异（*P* > 0.05）；良性病变的中转开胸率高于恶性肿瘤中转开胸率（*P* < 0.05）；病理分期为Ⅲa期的中转开胸率明显高于Ⅰ期、Ⅱ期的中转开胸率（*P* < 0.05）。见表 1。

**表 1:** 患者一般临床特征及手术病理资料 The general clinical characteristics and surgical pathology data of the patients

Clinical characteristics	VATS conversion (*n*=83)	VATS (*n*=1, 267)	*P*
Gender			0.243
Male	46 (55.42%)	789 (62.27%)	
Female	37 (44.58%)	478 (37.73%)	
Age (yr)			0.645
≥65	35 (42.17%)	501 (39.54%)	
< 65	48 (57.83%)	766 (60.46%)	
Smoking habit			0.560
Yes	49 (59.04%)	791 (62.43%)	
No	34 (40.96%)	476 (37.57%)	
Comorbidity			0.861
Emphysema	9 (10.84%)	149 (11.76%)	
Old tuberculosis	13 (15.66%)	225 (17.76%)	
No	61 (73.49%)	893 (70.48%)	
Tumor size (cm)	3.24±1.34	3.31±1.29	0.633
Histopathology			0.031
Malignant tumor	73 (87.95%)	1, 194 (94.24%)	
Benign lesion	10 (12.05%)	73 (5.76%)	
Pathological stage^*^	72 (86.75%)	1, 157 (91.32%)	< 0.001
Ⅰ	22 (26.51%)	654 (51.62%)	
Ⅱ	18 (21.69%)	305 (24.07%)	
Ⅲa	32 (38.55%)a, b	198 (15.63%)	
^*^72 primary lung cancer cases were included in the conversion to thoracotomy group and 1, 157 primary lung cancer cases in the VATS lobectomy group in the study. Comparison between the groups showed that the rate of conversion to thoracotomy group in stage Ⅲa and stage Ⅰ was ^a^*P* < 0.05; Compared with stage Ⅲa and stage Ⅱ, the conversion rate was ^b^*P* < 0.05. VATS: video-assisted thoracic surgery.			

### 2.2 不同肺叶切除术中转开胸率比较

联合肺叶切除术的中转开胸率明显高于单肺叶切除术（*P*=0.001）；左肺上叶肺叶切除术的中转开胸率显著高于其他单肺叶切除术（*P* < 0.001）；右肺中叶肺叶切除术的中转开胸率低于其他单肺叶切除术（*P*=0.049）。见表 2。

**表 2:** 不同手术方式中转开胸率比较 Comparison of the conversion rates of different surgical sites in the conversion to thoracotomy group

Surgical site	Total	VATS conversion	VATS	*P*
Combined lobectomy *vs* single lobectomy				0.001
Combined lobectomy	59	10 (16.95%)	49 (83.05%)	
Single lobectomy	1, 291	73 (5.65%)	1, 218 (94.35%)	
Left upper lobectomy *vs* other single lobectomy				< 0.001
Left upper lobectomy	301	32 (10.63%)	269 (89.37%)	
Other single lobectomy	990	41 (4.14%)	949 (95.86%)	
Right middle lobectomy *vs* other single lobectomy				0.049
Right middle lobectomy	119	2 (1.68%)	117 (98.32%)	
Other single lobectomy	1, 172	71 (6.06%)	1, 101 (93.94%)	

### 2.3 中转开胸组原因分析

术中中转开胸主要原因为血管损伤、淋巴结干扰、胸腔致密粘连等。中转开胸原因中肺动脉出血最为多见（31.33%），所有患者术中出血、淋巴结干扰、胸腔致密粘连等情况均经过手术成功处理，未出现严重并发症。见表 3、图 1。

**表 3:** 胸腔镜肺叶切除中转开胸的原因 Reasons for conversion to thoracotomy in VATS lobectomy

Reasons	Data
Vascular injury	32 (38.55%)
Pulmonary artery injury	26 (31.33%)
Pulmonary vein injury	2 (2.41%)
Azygos vein injury	2 (2.41%)
Superior vena cava injury	2 (2.41%)
Lymph node interferencea	22 (26.51%)
Dense adhesion	14 (16.87%)
Others	15 (18.07%)
Blood vessel stump bleeding	2 (2.41%)
Lung nodule was not touched	2 (2.41%)
Incomplete interlobar fissure	5 (6.02%)
Pulmonary artery resection by mistake	1 (1.20%)
Unclear organizational structure	2 (2.41%)
Complicated condition	1 (1.20%)
Failed tracheal intubation	2 (2.41%)
^a^lymph node interference refers to tumor or lymph node invasion blood vessels andbronchus.	

**图 1:**
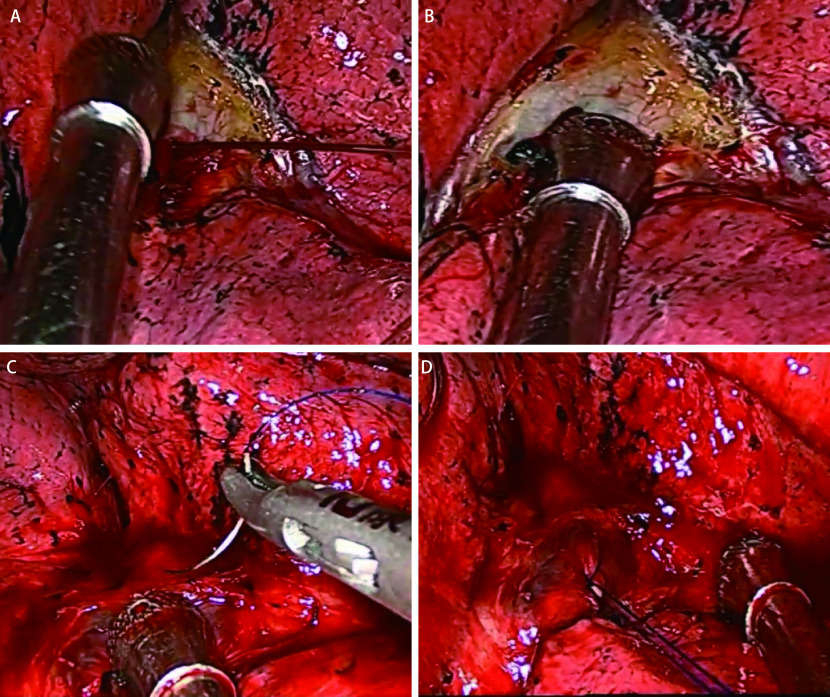
胸腔镜术中动脉出血及镜下止血典型病例。病例：女性，69岁，行胸腔镜右肺下叶切除手术，术中右肺动脉出血（A），吸引器压迫出血动脉, 控制出血并吸净出血区域（B），胸腔镜下用4-0 Proline线缝合止血（C、D）。 Typical cases of arterial bleeding and hemostasis in VATS lobectomy. Case: A 69-year-old female patient underwent right lower lobectomy by VATS. During the operation, the right lung artery showed hemorrhage (A); The aspirator was used to compress the bleeding artery, control the bleeding and clean the bleeding area (B); The bleeding was stopped by 4-0 Proline suture under VATS (C, D).

### 2.4 中转开胸组手术时间及术中出血量情况

中转开胸组总体手术时间为（236.99±66.50）min，总体失血量（395.85±306.38）mL。其中淋巴结干扰组手术时间为（322.50±22.68）min，长于其他原因中转开胸组（*P* < 0.05）；血管损伤组术中出血量为（560.94±361.84）mL，多于其他原因组（*P* < 0.05）。见表 4。

**表 4:** 中转开胸组手术时间及出血量情况（Mean±SD） Operation time and blood loss in the conversion to thoracotomy group (Mean±SD)

Reasons	Operation time (min)	Bleeding (mL)
Dense adhesion	240.05±62.75	359.09±293.84
Lymph node interference	322.50±22.68^a^	246.43±50.93
Vascular injury	210.84±58.21	560.94±361.84^b^
Others	206.43±44.95	225.71±77.43
Total	236.99±66.50	395.85±306.38
Compared with the other groups, the operative time was ^a^*P* < 0.05; Compared with the other groups, the amount of blood loss was ^b^*P* < 0.05.		

### 2.5 不同阶段VATS肺叶切除术中转开胸率比较

根据手术例数每450例为一组，将全组分为手术前期（前450例）、中期（中间450例）及后期（后450例）。比较发现随着时间的推移以及经验的积累，血管损伤例数逐步下降（*P*=0.045）。见表 5。

**表 5:** 各组VATS肺叶切除术中转开胸手术情况 Summary of conversion to thoracotomy in each group

Stage	Reasons				Conversion rate
	Vascular injury	Lymph node interference	Dense adhesion	Others	
Early	17 (3.78%)	10 (2.22%)	7 (1.56%)	5 (1.11%)	8.67%
Middle	9 (2.00%)	7 (1.56%)	5 (1.11%)	6 (1.33%)	6.00%
Late	6 (1.33%)	5 (1.11%)	2 (0.44%)	4 (0.89%)	3.78%
*P*	0.045	0.416	0.292^*^	0.817	0.009
^*^Using *Fisher's* exact test.					

## 3 讨论

自20世纪90年代初以后，胸腔镜肺叶切除术已逐步成为肺癌的标准治疗方法^[6]^。有研究^[7, 8]^表明，与传统开胸手术相比，胸腔镜手术具有创伤小、并发症少、住院时间短以及更好地保留肺功能等优点。尽管如此，胸腔镜手术也存在一定局限性，例如较小的手术切口及有限的手术角度会导致胸腔内操作困难，存在中转开胸的可能。多项研究^[9-11]^显示，胸腔镜肺叶切除手术中转开胸率为2.5%-28%。但随着胸腔镜技术水平的提升及设备的改进，本研究平均中转开胸率基本稳定于6%，同国外相关文献^[11, 12]^报道相仿。有研究^[13, 14]^显示，胸腔镜肺叶切除术中转开胸与患者高龄、肿瘤TNM分期、胸部纤维钙化淋巴结存在有关。本研究中的良性疾病多为炎性病变，由于长期慢性感染导致胸膜腔粘连、支气管壁与周围结构不清、淋巴结肿大钙化、支气管动脉迂曲等变化，从而增加了胸腔镜肺叶切除术手术难度和中转开胸风险。在恶性肿瘤患者中，分期较晚的患者往往伴随着肿瘤较大、淋巴结与周围组织界限不清等不利于胸腔镜手术操作的因素，因而，其中转开胸率也比较高。

多项研究结果^[5, 7, 8]^显示，血管损伤是胸腔镜肺叶切除术中转开胸最常见的原因，占所有中转开胸的30.4%-40%。本研究结果显示，血管损伤占所有中转开胸的38.55%。按照不同时段进行分组时，血管损伤与手术例数的增加呈负相关。推测原因如下：一方面，随着腔镜技术的提升，血管损伤发生率逐渐降低，另一方面术中某些血管损伤出血量较小，可在腔镜下进行夹闭或修补，随着经验的积累，以前在腔镜下不易控制的中等速度乃至高速度出血，后来也多能通过腔镜下阻断和缝合加以有效控制。我们的经验是，肺动脉损伤出血时，可以用胸腔止血钳夹闭出血部位或吸引器侧壁压迫出血部位，待出血在可控范围内，再于腔镜下进行止血（如图 1所示）；如出现血管残端出血，渗血速度较慢，可给予纱布压迫止血，必要时给予钛夹或Hemolock结扎夹夹闭残端止血；如出血动脉近端解剖距离不足时，可游离同侧肺动脉干，用无创血管钳阻断肺动脉干，待出血控制后，对出血血管进行修补。当出现拟切除的粗大肺静脉损伤出血时，可在局部压迫出血部位前提下，迅速游离出血血管近心端，必要时打开心包离断血管。如出血汹涌，应迅速压迫出血部位，尽力控制出血的同时紧急进行中转开胸处理。本研究同时发现，与胸腔镜单肺叶切除术相比，联合肺叶切除术中转开胸率较高，这可能与肿瘤分期较晚有关。而在单肺叶切除术中，研究发现胸腔镜右肺中叶切除术中转开胸率最低（1.68%），左肺上叶切除术中转开胸率最高（10.63%）。其原因在于左肺上叶动脉变异最大，分支最多，且左肺上叶分支动脉前方有气管遮挡。如按照传统的开胸手术方式操作先离断动脉再切断气管，在游离左肺上叶动脉时，由于手术操作空间较局限，可能会因为过度牵拉而将左肺动脉主干分支夹角处撕裂引起出血；如果按照胸腔镜单向式手术方式操作先切断气管再离断动脉，如气管后方与动脉粘连紧密，过度将肺向上方提拉，同样可以造成动脉出血，且此处出血比较汹涌，增加了中转开胸的可能。为了进一步降低因术中出血而中转开胸的比例，我们的经验如下：首先，术前仔细阅读患者计算机断层扫描（computed tomography, CT）、磁共振成像（magnetic resonance imaging, MRI）等影像学资料，充分了解肿瘤与周围血管关系，必要时术前行血管造影评估血管受侵情况，严格掌握胸腔镜手术适应证，如手术无法切除肿块，可考虑实行新辅助化疗，待肿块缩小后再考虑手术治疗；术中仔细辨认血管壁及周围组织，尽可能在血管鞘内进行游离血管。对于肿瘤侵犯血管，预计有可能损伤大出血时，可先游离血管上、下方，并放置硅胶管棉线套带，以防止一旦出血时可有效进行阻断，待出血减慢或控制后，再进行安全的修补；如遇到病灶较大，肿瘤外侵较重，如肿瘤侵及纵隔大血管、肺门结构等，需结合术者自身手术水平而定，若无法处理，及时中转开胸。

淋巴结嵌顿、无法分离也是引起胸腔镜肺叶切除术中转开胸的常见原因^[14]^。本研究结果显示，淋巴结干扰中转开胸组手术时间高于其他原因组，其原因可能是淋巴结与动脉、气管粘连紧密，导致分离困难，此类情况通常是由于肺部长期慢性炎症引起的肺部淋巴结胶原化甚至钙化造成。在本研究中，21例淋巴结嵌顿中转开胸患者中仅有2例是由于转移淋巴结侵犯肺血管引起，其余19例中转开胸患者与肺部良性疾病、慢性炎症有关。在进行胸腔镜肺叶切除术时，当遇到钙化淋巴结与动脉、支气管粘连严重，无法分离时，由于这种淋巴结通常不是转移的淋巴结，可通过锐性结合顿性的方法去除淋巴结内钙化部分，然后将其与血管、支气管一同处理。当遇到非钙化淋巴结与血管难以分离，无法打开动脉鞘游离动脉时，应做好中转开胸的准备。Samson等^[15]^报道通过胸部CT检查来对胸部淋巴结钙化的部位及程度进行评分，根据评分数据预测胸腔镜肺叶切除术中转开胸的可能。这对指导我们在术前选择合适的胸腔镜手术患者，术中降低因淋巴结干扰而中转开胸等方面可能具有一定临床意义。

胸腔粘连也是引起胸腔镜肺叶切除术中转开胸的原因之一。文献^[16-18]^报道，因胸膜粘连导致中转开胸占所有中转开胸的比例在7.7%-20%之间。本研究结果显示，胸膜粘连占所有中转开胸的16.87%。慢性胸膜炎是引起胸腔粘连的主要原因，其多数由肺结核引起，但结核性胸膜炎引起的胸腔粘连临床症状并不典型，受到当地经济及教育水平的影响，部分患者只有通过胸部手术时才被发现。本研究认为，对于大范围的致密粘连，可用双手手指顿性分离胸膜腔，直至两指尖打通后，伸入胸腔镜镜头进行分离，必要时也可通过适当延长切口及增加操作口数量进行粘连分离，大多能够完成分离。若镜下分离困难时，特别是胼胝样粘连，或二次手术患者，应酌情尽早考虑开胸手术。

胸腔镜肺叶切除术中转开胸是由多种因素共同参与的结果。本研究显示，在开展胸腔镜肺叶切除术前期，术中发生血管损伤出血，由于助手不能很好地配合术者来完成腔镜下血管缝合，被迫中转开胸；而在开展胸腔镜肺叶切除术后期，随着助手与术者配合愈发默契，因血管损伤出血而中转开胸的比例有所降低。胸腔镜手术开展前期阶段，胸腔粘连中转开胸的比例较高，但是随着胸腔镜肺叶切除术例数的增多以及经验的积累，其中转开胸的比例降低，因此胸腔粘连并非是胸腔镜肺叶切除术禁忌证。本组手术主要以单操作孔完成，部分手术采用纯单孔完成，当术中遇到出血、手术难度较大时，会采用三孔法完成，由于在本研究中三种手术方式并没有发现差异，因此没有再单独对这三种手术方式进行比较和讨论。必须指出的是本研究中的手术团队是从零基础开始，整个团队的磨合和培养经过了较长时间的学习过程。如果一个术者在当前良好设备及成熟团队等优势条件下经过悉心培养，其成长所需的时间可能要比本研究明显缩短。本研究中转率稍高的另一个原因是有19例经胸腔镜初步探查后尚未进行肺门结构的游离，而随即中转开胸的患者，此类患者严格意义上讲，并不在中转开胸的范围之内，但为了保证资料的完整性，仍将其纳入此研究中。

本研究也具有一定的局限性：①本研究为单手术组的回顾性研究，其结果可能会因为手术者个人对胸腔镜手术的认识、手术指征的把握、手术操作的技巧乃至整个医学理论认识的局限性而产生一些偏差；②每一例胸腔镜手术中转开胸往往都是多种因素共同作用的结果，本研究试图探寻导致中转开胸的主要原因，以降低中转开胸的风险；③本研究时间跨度大，因胸腔镜技术飞速发展，包括打孔方式、操作手法、手术器械、腔镜设备甚至手术理念都在发生日新月异的变化，这些变化也可能会对本研究的结果产生一定的影响。

综上所述，虽然胸腔镜肺叶切除术日益常态化，但中转开胸仍然难以避免。就我院单手术组研究结果表明，血管损伤、淋巴结干扰、胸腔致密粘连仍是常态下胸腔镜肺叶切除术中转开胸的主要原因。胸腔镜肺叶切除术中转开胸的主客观原因较复杂，正在常态下进行胸腔镜手术学习的临床工作者，需选择合适的手术患者，准确把握中转开胸的手术指征，以提高常态下胸腔镜肺叶切除的安全性。
